# Contrasting Patterns of Larval Mortality in Two Sympatric Riverine Fish Species: A Test of the Critical Period Hypothesis

**DOI:** 10.1371/journal.pone.0109317

**Published:** 2014-10-09

**Authors:** Nicole McCasker, Paul Humphries, Shaun Meredith, Nicholas Klomp

**Affiliations:** 1 School of Environmental Sciences, Charles Sturt University, Albury, New South Wales, Australia; 2 Institute of Land, Water and Society, Charles Sturt University, Albury, New South Wales, Australia; 3 Aquatic Environment Branch, Department of Fisheries, Cloisters Square, Western Australia, Australia; 4 University of Canberra, Bruce, Australian Capital Territory, Australia; Institute of Marine Research, Norway

## Abstract

Understanding the causal mechanisms that determine recruitment success is critical to the effective conservation of wild fish populations. Although recruitment strength is likely determined during early life when mortality is greatest, few studies have documented age-specific mortality rates for fish during this period. We investigated age-specific mortality of individual cohorts of two species of riverine fish from yolksac larvae to juveniles, assaying for the presence of a “critical period”: A time when mortality is unusually high. Early life stages of carp gudgeons (*Hypseleotris* spp.) and unspecked hardyhead (*Craterocephalus stercusmuscarum fulvus*)—two fishes that differ in fecundity, egg size and overlap between endogenous and exogenous feeding—were collected every second day for four months. We fitted survivorship curves to 22 carp gudgeon and 15 unspecked hardyhead four-day cohorts and tested several mortality functions. Mortality rates declined with age for carp gudgeon, with mean instantaneous mortality rates (-*Z*) ranging from 1.40–0.03. In contrast, mortality rates for unspecked hardyhead were constant across the larval period, with a mean -*Z* of 0.15. There was strong evidence of a critical period for carp gudgeon larvae from hatch until 6 days old, and no evidence of a critical period for unspecked hardyhead. Total larval mortality for carp gudgeon and unspecked hardyhead up to 24 days of age was estimated to be 97.8 and 94.3%, respectively. We hypothesise that life history strategy may play an important role in shaping overall mortality and the pattern of mortality during early life in these two fishes.

## Introduction

One of the primary challenges in ecology is to understand the intrinsic and extrinsic factors that influence survival in the early life of animals and plants, when mortality is typically greatest [1]–[3]. Ontogeny involves major physiological, behavioural and morphological changes and so mortality is unlikely to be constant during this time. Fish - which typically produce hundreds, thousands and sometimes many millions of young - provide the most extreme examples of mortality of any vertebrate [4].

The critical period hypothesis proposed for fish states that there is a time during early life when mortality is unusually high, and it is at this stage that the longer-term survival rates of the cohort, or year-class strength, is determined [5], [6]. More specifically, the transition from endogenous to exogenous feeding has been traditionally heralded as the ‘critical period’ for fish [5], though more recently it has been suggested that other critical periods may exist within the early life stages, including the embryo stage [7], [8], the newly hatched pre-feeding stage [9], and juvenile metamorphosis [10], [11].

Critical periods in fish ontogeny have been the subject of many investigations over the last century, however, conclusions as to both their existence and importance in determining year-class strength remain equivocal [6], [12]. This is because of methodological and analytical limitations that include pooling of data across large spatial scales, the effects of immigration and emigration, the patchy distribution of young over temporal and spatial scales, and a lack of mortality estimates that encompass the entire larval period [13], [14]. Analytical limitations relate to the absence of comparisons of alternative models that discriminate between constant and varying mortality (but see [15]–[17]).

One of the key methodological limitations in the way studies have assessed mortality in larval fish has been the use of catch curves that incorporate abundance-at-age data collected from broad spatial scales, with large temporal resolution [1], [13], [18], [19]. Sampling at large spatial scales (i.e. 50–100 km) usually coincides with large temporal resolution, which is likely to be insufficient to detect brief fluctuations in mortality related to specific developmental stages. Further, sampling at large temporal resolution (weeks to months) usually results in age-abundance data being averaged across cohorts, producing an artificially smoothed survival curve that bears little relevance to the real mortality risks experienced by individual cohorts. Overall, sampling at inappropriate scales will mean the loss of information at the level that mortality is likely to occur.

One key analytical limitation in the way studies have assessed mortality in larval fish is that alternative mortality models are rarely tested. There is general agreement that a critical period can be defined as a period of higher than average instantaneous mortality. However, the existence of critical periods has been inferred largely from subjective estimates of the goodness-of-fit of exponential decay models (i.e. constant mortality) to abundance-at-age data, without exploring alternative models [20], [21]. A superior approach would be to compare abundance-at-age data against two alternative models for mortality: one which assumes that mortality is constant irrespective of developmental stage or age (i.e. absence of critical period), and another that allows for mortality rates to change with developmental stage or age (i.e. presence of critical period). To date, alternative hypothesis testing has rarely been conducted (but see [15], [17], [22]).

The present study aims to redress these limitations by investigating the presence or absence of a critical period for two protracted-spawning riverine fishes: (a) in a section of river where immigration and emigration are limited; (b) at temporal and spatial scales relevant to mortality of small organisms that show limited movement; (c) by assessing multiple cohorts over several months; and (d) by comparing alternative mortality models. We use carp gudgeon (*Hypseleotris* spp.: Eleotridae) and unspecked hardyhead (*Craterocephalus stercusmuscarum fulvus*: Atherinidae) as model species to investigate possible differences in the rates of mortality during the larval phase. Both species are small, they are protracted spawners, breed by age 1 and only live for at most 3 years and would be classified as ‘opportunistic’ species (Table 1, [23], [24]). However, carp gudgeon has more altricial traits: it produces relatively large numbers of small eggs and exogenous feeding commences only after the depletion of yolk reserves [25], [26]. By contrast, unspecked hardyhead has more precocial character traits: it produces relatively fewer, larger eggs and begins exogenous feeding before the depletion of yolk reserves [27].

**Table 1:** Life history traits of carp gudgeon and unspecked hardyhead.

Life history traits	Carp gudgeon	Unspecked hardyhead	References
Adult size (mm)	40	50–60	[59]
Longevity (years)	2–3	2–3	[59]
Larval duration (d)	<30 days	<30 days	[26], [27]
Fecundity (eggs.batch^−1^)	1000–2000	20–100	[60], [27]
Egg size (diameter, mm)	0.4–0.5	1.3–1.7	[60], [27]
Egg type	demersal, adhesive	demersal, adhesive	[26], [27]
Size at hatch (mm)	1.8–2.1	5	[60]
Age at 1^st^ feeding (d)	3–4	5–6	[49], [27]
Approx. time to hatching (h)	47–53	96–216	[49]
Endogenous/exogenous overlap (days)	0	3–5	[25]–[27]

## Materials and Methods

This study was carried out in strict accordance with the recommendations set out in the Australian Code of Practice for the Care and Use of Animals for Scientific Purposes. The protocol was approved by the Animal Ethics Committee of Charles Sturt University (Permit numbers 05/062 and 06/107). Fish larvae were collected under the Victorian Fisheries Research Permit no RP584, and access to field sites in the Murray Sunset National Park was permitted under National Parks Research Permits 10003422, 10004266 and 10004803.

### Study species

The two species used for this study were carp gudgeon (*Hypseleotris* spp.) and unspecked hardyhead (*Craterocephalus stercusmuscarum fulvus*). Carp gudgeon is a species complex, comprising a number of hybrids, which are morphologically indistinguishable [28]. Carp gudgeon are small, zooplanktivorous fish which grow to 40 mm, reach sexual maturity in their first year, and live for a maximum of 3 years (Table 1). At hatch, larvae are 1.5–2.1 mm and poorly developed, with unpigmented eyes, jaws incapable of feeding, and pectoral fins not yet formed. There is no overlap in transition from endogenous to exogenous feeding [25], [29]. The unspecked hardyhead is a recognised sub-species of one of seven atherinid species occurring in the river systems of eastern Australia. Unspecked hardyhead produce larger eggs (1.3–1.7 mm) and are less fecund than carp gudgeon (Table 1). Newly hatched larvae are well developed (5 mm TL), have pigmented eyes, ossified and fully functioning jaws, and capable of free-swimming [29]. In contrast to carp gudgeon, hardyheads have a transitionary overlap between endogenous and exogenous feeding of 3–5 days (Table 1).

### Field collections

**Figure 1:**
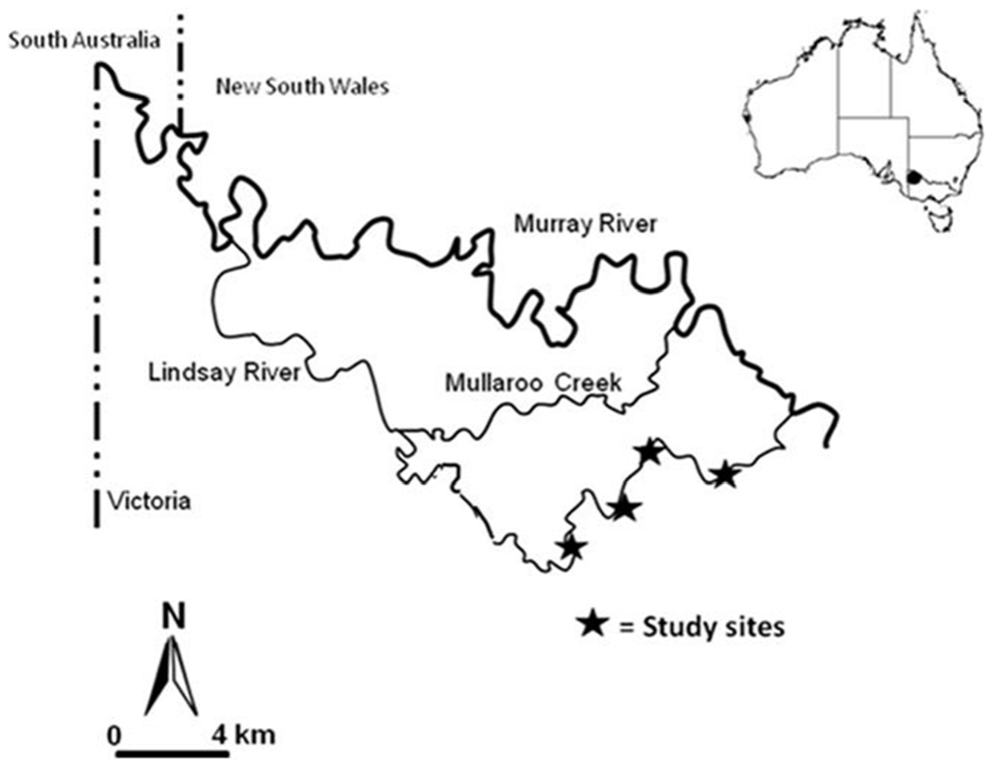
Map of the study area; Lindsay River, Victoria, Australia.

Carp gudgeon and unspecked hardyhead larvae were collected from four 500 m reaches of the Lindsay River, a 32 km anabranch of the River Murray, in south-east Australia (Figure 1). The Lindsay River is situated within the semi-arid, spring-winter rainfall zone of the Murray-Darling Basin, Australia, where it leaves the Murray River 8 km upstream of Lock and weir 7 (one of 13 weirs located along the Murray River), and travels south-west until it re-enters the main channel just above Lock and weir 6, near the borders of three states. The section of the Lindsay River studied is lentic; with occasional flow occurring, but at very slow current velocities (0–1 cm.s^−1^). The main channel of the Lindsay River is approximately 21 km long, 20 m wide and 1.5 m deep. The lentic nature of the upper Lindsay River makes it an ideal environment to study mortality dynamics in larval fish populations as larval fish are poor swimmers and unlikely to move out of this system. By studying the dynamics of the early life history of small fish species in a system where flow is minimal, it can be assumed that immigration or emigration are negligible, and so population changes are most likely because of births and deaths.

Fish larvae were sampled from the four reaches at night (approx. 2300–0200 h) using modified quatrefoil light traps. Three light traps were deployed for 1.5 h at each reach on every second night from 8 October 2005 to 8 February 2006. Samples were preserved in 90 % ethanol and taken back to the laboratory for further processing. There, all larval and juvenile fish were identified and measured (TL) with a dissecting microscope and ocular micrometer to 0.1 mm and classified as being yolksac larvae, protolarvae, flexion, post flexion, or juvenile/adults, following Kelso and Rutherford (1996) [30]. We considered larvae to have reached juvenile metamorphosis when individuals possessed the full complement of adult characters, including scales, fins, rays and spines, had evidence of segmentation of soft rays, and fin folds had been lost [30].

The spatial scale of the study (10–12 km) was small compared to that commonly used in larval mortality field studies (50–100 km, [31], [32]). The decision to pool larvae across the four reaches (adjacent reaches were 3 km apart), rather than derive mortality rates for each reach was made because, although it is unlikely that movement of larvae in and out of the study area (most downstream to upstream sites) would take place within 2 days, it could not be assumed that movement in and out of the individual study reaches would be negligible during the study period. Taking this into consideration, and because environmental conditions were similar across the study reaches, we considered it was more appropriate to estimate mortality rates from the study area as a whole rather than calculate mortality rates for each study reach.

### Aging techniques and otolith analysis

Estimates of the ages of larvae were determined using age-length regression equations, with ages determined using otolith analysis. A total of 240 sagittal otoliths were removed from monthly sub-samples of usually 30 carp gudgeon and 30 hardyhead larvae, representing approximately 5% of fish collected. The otoliths were mounted, sulcus side down, on glass microscope slides with the epoxy resin Crystalbond (Ted Pella Inc, USA). Ages of the larvae were determined by counting the number of increments at 400× or 1000× under bright-field illumination with a compound microscope. The number of increments of each otolith was counted twice by the primary reader. Verification of the counts was conducted by a second reader, and only those counts that were in agreement with both readers were included in the analysis; 227 otoliths in all.

The deposition of daily growth increments in otoliths was validated for both carp gudgeon and unspecked hardyhead ([33]; and see Appendix S1). The first growth increment was assumed to coincide with hatch date. While this was not validated, otolith analysis of the smallest, least developed yolksac larvae revealed a maximum of only one otolith increment, and all yolksac larvae had increments, indicating that deposition of the first increment was almost certainly a hatch check, and unrelated to first feeding.

To take into account the influence of temperature on growth rates, age-length relationships were established for each month of the study. Month was found to be more than adequate, because age-length relationships differed only marginally (<2 days) between consecutive months. Growth estimates (mm.day^−1^) were calculated by fitting alternative growth models to the data and using the model of best fit. Three growth functions were fitted to the data: linear, von Bertalanffy, and Gompertz models. A total of 105 carp gudgeon and 122 hardyhead larvae/early juveniles were aged and used to construct monthly age-length relationships. Akaikie's Information Criterion (AIC) was used to determine the most appropriate growth model for each month. Confidence intervals (95%) were computed for each monthly growth function using standard regression techniques to establish the variation in length-at-age for each species and month. For each species, monthly growth equations were re-arranged and solved for age (days). From these, the age of each carp gudgeon and unspecked hardyhead collected during the study was calculated. Residuals of predicted age were plotted to investigate any error associated with calculating age from length using the models of best fit. To take the age-estimation error into account, larval abundances for each cohort were grouped into 2-day age class bins (e.g., 1-2 day, 3–4 day old larvae).

AIC scores indicated that carp gudgeon growth was best predicted in November and January by von Bertalanffy, in October by Gompertz and in December by Linear models. All growth models, when resolved, predicted length from age well (carp gudgeon; R^2^ > 0.95, unspecked hardyhead; R^2^ > 0.85 (Appendix S2). Unspecked hardyhead growth was best predicted by Gompertz (November, January) and linear models (October, December); von Bertallanfy models could only be resolved on one occasion. Regardless, comparisons between the plots of predicted age and their associated residuals indicated minimal difference in error among the three alternative growth models, when they could be resolved (Appendix S2). Because there was minimal difference in the error associated with the use of the Gompertz model and the model of best fit as determined by AIC, and because the Gompertz model is frequently used to describe growth during the larval stages of freshwater and marine fish [34], the Gompertz model was used to determine age from length for all months and species. The majority of age-length residuals fell within the 95% confidence limits of ± 3.9 days, so the decision was made to investigate mortality rates of cohorts of larvae born within 4 days of each other. With the lengths of all larvae measured, and their associated ages estimated, ‘hatch dates’ were then assigned for each carp gudgeon and unspecked hardyhead larvae, determined as the date of capture minus estimated age.

### Ontogenetic development

The rate of ontogenetic development in carp gudgeon and unspecked hardyhead was determined by calculating the length of time larvae spent in each major development stage (YS, yolksac larvae; PL, protolarvae; F, flexion; PF, post flexion; and ML, metalarvae). Box-plots of age for each stage and month were constructed to determine the monthly median length of time (days) larvae spent within each major developmental stage. Larval stage duration (LSD) was taken as the number of days between the 25^th^ and 75^th^ percentiles. The range of ages for each stage was calculated for each month separately. The overall time taken to reach metamorphosis for each month was calculated by adding the time taken to complete each developmental stage.

### Abundance-at-age survival curves

Mortality estimates were calculated by constructing survival curves using log_e_(x+1) transformed abundance-at-age data for each cohort of larvae born within 4-5 days of each other. The rate of decline along the survival curve was calculated for each cohort at 2 day intervals, commencing from 1-2 day old yolksac larvae, up until the time that cohorts metamorphosed into juveniles. For carp gudgeon, corresponding total lengths (TL) for newly hatched larvae and recently metamorphosed juveniles were approximately 3.2 and 9.5 mm respectively, and metamorphosis was generally achieved by 24 days. For unspecked hardyhead, corresponding total lengths for newly hatched larvae and recently metamorphosed juveniles were 6.0 and 11.0 mm, respectively. Metamorphosis was generally achieved by 26 days.

To determine if there was a period during the larval phase of carp gudgeon and unspecked hardyhead at which higher than average instantaneous mortality rates (Z) occurred, three alternative models (linear, Weibull and asymptotic) were compared against abundance at age data for each cohort. The linear function (often presented as an exponential function on untransformed data), is the most commonly used survival curve to estimate mortality rates in fish populations. Best fit of the linear equation indicates that mortality rates are constant across larval age, and so there is no evidence of a critical period. The Weibull function is a commonly employed ‘hazard function’ (measuring the mortality rate as a function of age) in general survival analyses [35]. It is rarely used in fisheries ecology (but see [36]), but, unlike the linear function, the Weibull function provides a better fit for populations with a temporally variable mortality rate. The asymptotic function behaves similarly to that of the Weibull, but best describes populations with a monotonically decreasing mortality rate. Thus, in this study, best fit of either the Weibull or asymptotic functions indicated that cohorts experienced higher mortality rates at some stage, and thus provide evidence of the existence of a critical period. The alternative mortality models were compared using AICc values. A difference of 3 or more between the AICc values was used to distinguish differences between models and the best fit was determined by the lowest AICc score (see Appendix S3 for model equations and parameters). From this, instantaneous daily mortality rates could be estimated for each cohort as the slope of the descending portion of the log_e_(x+1) transformed curve.

### Mortality rates

Survival curves were produced to calculate overall mortality rates for each cohort, as well as 2-d age-specific mortality rates for carp gudgeon and unspecked hardyhead. The fitting of survival curves was limited to the ages of larvae considered fully recruited to the gear, as represented by the descending right-hand portion of the abundance-at-age data. Visual inspection of unspecked hardyhead abundance at age data indicated that larvae aged 1-2 days old were underrepresented by the sampling gear, and thus were excluded from the survival curve analysis. For several cohorts, survival curves could not be produced, as model parameters would not converge. In total, 22 carp gudgeon and 15 unspecked hardyhead cohorts were followed. Several estimates of age-specific mortality were calculated, including daily mortality rates, and instantaneous mortality rates. Daily mortality rate (M_daily_, % loss.day^-1^) was calculated as the proportion of individuals lost from the previous age class:
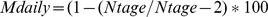
where Nt_age_ = abundance of larvae at age x, and Nt_age-2_ is the abundance of larvae 2 days prior.

The instantaneous daily mortality coefficient (*Z*) for each cohort was calculated using the derivative form of the equation which best described each cohort's survival curve.

Estimating mortality rates from abundance assumes that i) sampling gear is not size selective, and ii) emigration from and immigration to the study reaches is negligible. To test the first assumption, we conducted a mesocosm experiment to identify any size selectivity of the light traps. Light traps successfully caught 100% of all carp gudgeon and unspecked hardyhead larvae in the mesocosms, with size-selectivity of the gear only occurring at much larger sizes when individuals were well into the juvenile and adult stages (Appendix S4). The second assumption is also likely met because during the study period the Lindsay River had minimal flow, which would have meant that larvae could not have moved out of the study area passively and it is highly unlikely that they would have done so actively, as they are not known to disperse as larvae. All statistical analyses were performed in R.2.15 [37].

## Results

### Ontogenetic development and time to metamorphosis

**Figure 2:**
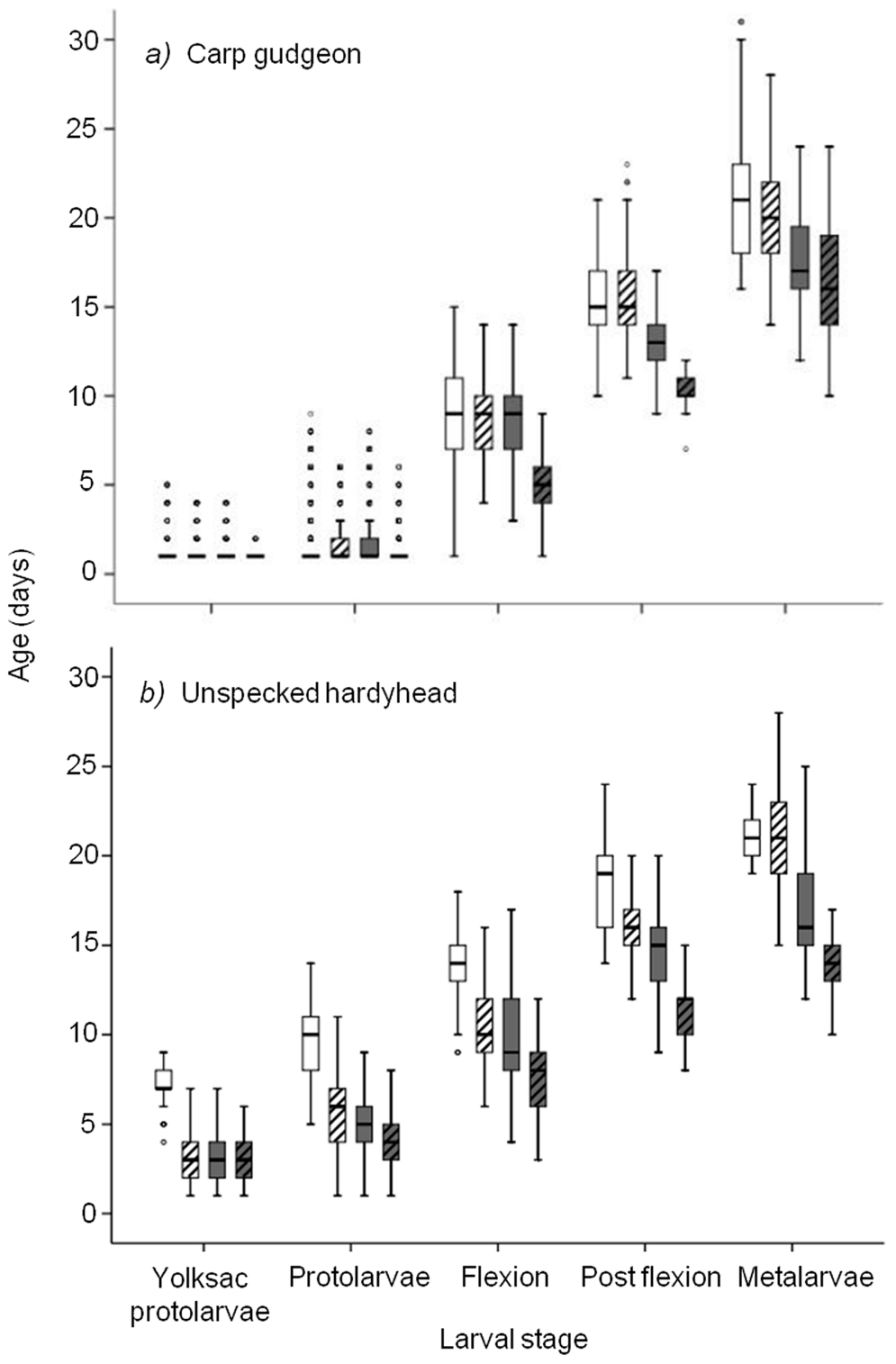
Monthly frequency box-plots of age (days) of each key developmental stage for a) carp gudgeon and b) unspecked hardyhead. October born larvae (white), November born larvae (white-striped), December born larvae (grey) and January born larvae (grey-striped).

Despite there being little difference in growth rates of larvae over the spawning season, the length of larval duration for carp gudgeon and unspecked hardyhead decreased with the progression of spawning season (Figure 2). This trend was more consistent for unspecked hardyhead than for carp gudgeon. For example, in October and November, the majority of hardyhead larvae had reached juvenile metamorphosis by 21 and 22 days, respectively, whereas in December, metamorphosis was reached by 19 days, and January it was reached by 15 days. By comparison, in October, most carp gudgeon larvae had reached juvenile metamorphosis by 23 days, and in November, December and January it took 21, 20 and 19 days, respectively. The median age of carp gudgeon with yolksac reserves still present was 1 day, while the median age of unspecked hardyhead with yolksac reserves observed ranged from 4–7 days.

### Evidence of a critical period

**Figure 3:**
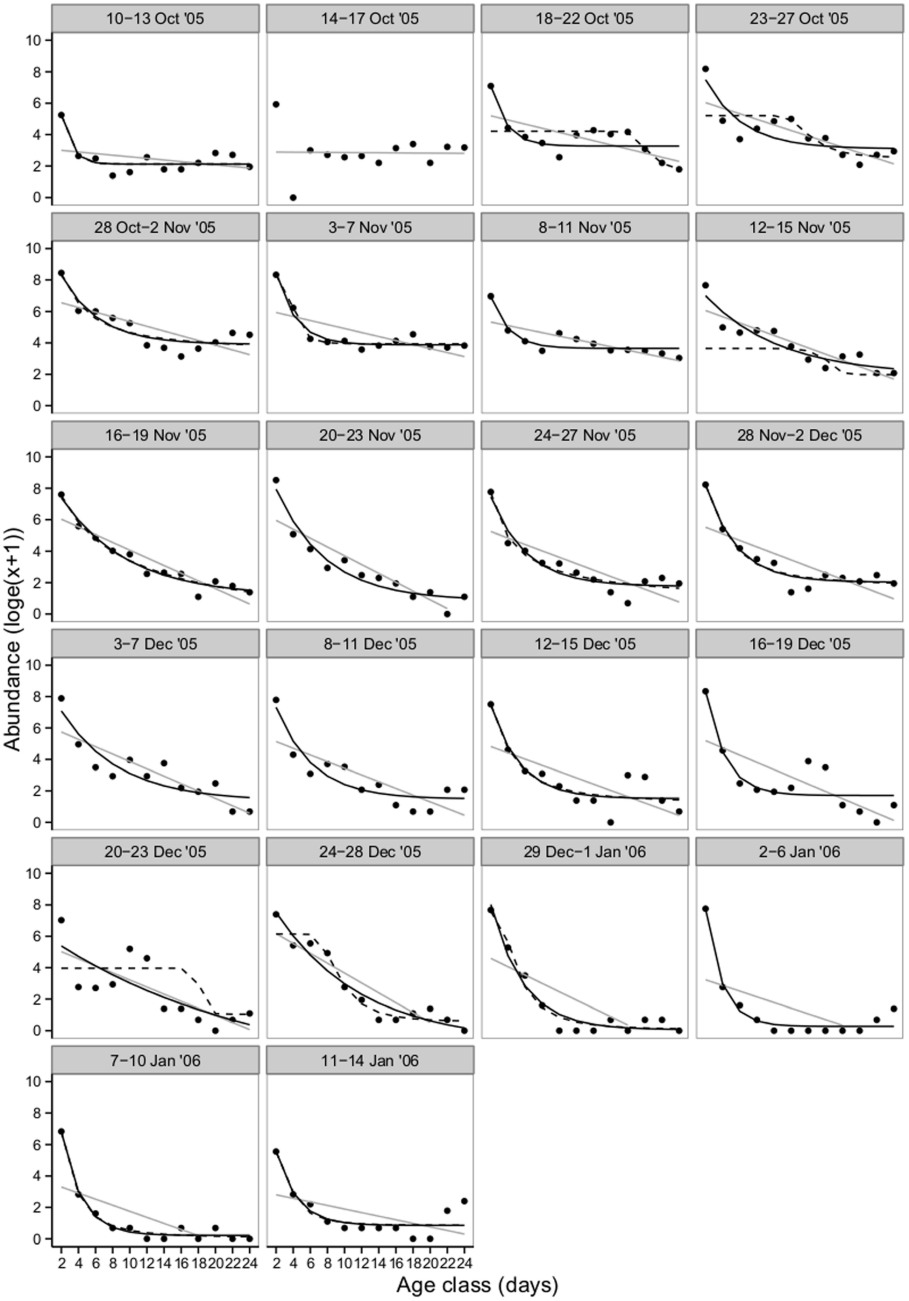
Individual cohort survivorship curves for carp gudgeon larvae in the Lindsay River between October 2005 and February 2006. Data log_e_ (x+1) transformed. Black dotted line  =  Weibull function (non-constant mortality model), black solid line  =  asymptote function (non-constant mortality model), and grey solid line  =  linear function (constant mortality model). Age class (days) represent 2 day groupings of larvae (e.g. 2 = 1-2 day old larvae, 4 = 3–4 day old larvae etc).

**Figure 4:**
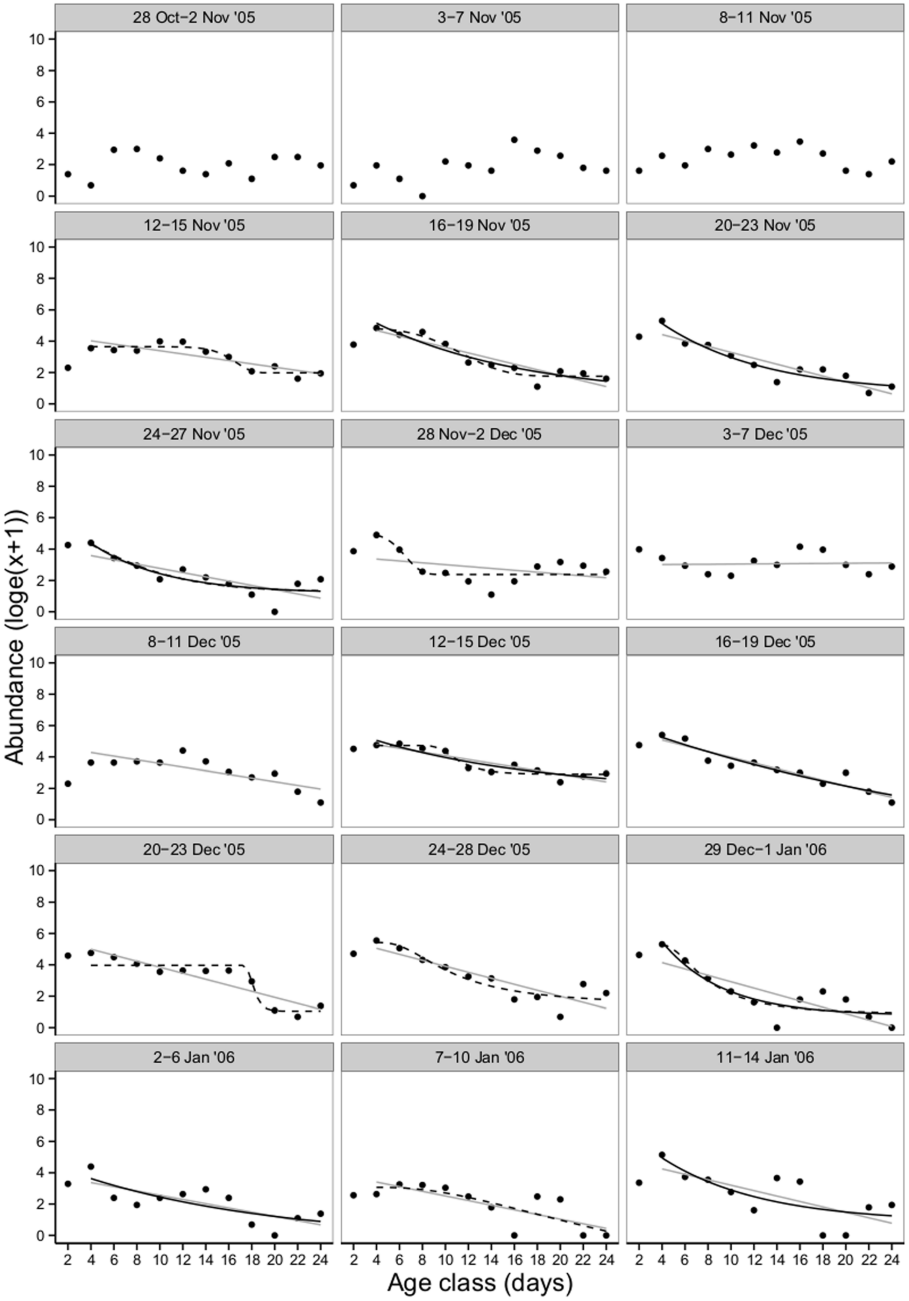
Individual cohort survivorship curves for unspecked hardyhead larvae in the Lindsay River between October 2005 and February 2006. Data log_e_ (x+1) transformed. Black dotted line  =  Weibull function (non-constant mortality model), black solid line  =  asymptote function (non-constant mortality model), and grey solid line  =  linear function (constant mortality model). Age class (days) represent 2 day groupings of larvae (e.g. 2 = 1–2 day old larvae, 4 = 3–4 day old larvae etc).

A total of 64 563 carp gudgeon and 9 405 unspecked hardyhead larvae and early juveniles were collected from the Lindsay River between 8 October 2005 and 8 February 2006. Twenty-two carp gudgeon cohorts that hatched between 10 October 2005 and 28 January 2006, and 15 unspecked hardyhead cohorts that hatched between 12 November 2005 and 28 January 2006 were followed throughout their larval development until 24 days old. From 22 carp gudgeon cohorts, 21 could be tested for both constant and non-constant mortality (Table 2). AICc scores indicated that 18 of the 21 cohorts were best fitted by the Weibull or asymptote algorithms. Mortality rates for carp gudgeon were therefore not constant with age, but were found to be greatest during the first 6 days post hatch (Figure 3). This time of high mortality generally encompassed the yolksac protolarvae stage through to the end of the protolarvae period, after which mortality rates remained relatively low and constant until juvenile metamorphosis. From the 15 unspecked hardyhead cohorts, 13 could be tested for both constant and age-specific mortality (Table 2), and of these, AICc scores indicated that mortality was constant across for all cohorts during the larval phase (i.e., the linear model provided the best fit, Table 2, Figure 4).

**Table 2:** AICc results for the alternative models of mortality during the larval phase of carp gudgeon and unspecked hardyhead for each 4d cohort; linear (constant mortality (Z)), asymptote (non-constant mortality (Z)) and Weibull (non-constant mortality (Z)) functions.

Cohort No.		Constant Z		Non-constant Z					
		Linear		Asymptotic		Weibull			
	Hatch date	AICc	SE	AICc	SE	AICc	SE	ΔAICc	CP
*carp gudgeon*									
c1	10–13 Oct	38.7	0.98	**24.7**	0.49	30.4	0.52	14.0	Y
c2	14–17 Oct	47.1	1.39	–	–	–	–	–	?
c3	18–22 Oct	39.8	1.02	**38.3**	0.87	50.6	1.22	1.5	N
c4	23–27 Oct	**39.7**	1.02	40.3	0.94	51.7	1.27	–0.6	N
c5	28–2 Oct	40.4	1.05	**30.9**	0.64	37.4	0.70	9.6	Y
c6	3–7 Nov	41.2	1.09	**16.4**	0.35	**16.4**	0.29	24.7	Y
c7	8–11 Nov	30.7	0.70	**22.7**	0.45	–	–	8.1	Y
c8	12–15 Nov	32.4	0.75	30.6	0.63	**17.5**	0.31	15.0	Y
c9	16–19 Nov	33.7	0.80	**20.2**	0.41	25.2	0.42	13.5	Y
c10	20–23 Nov	41.1	1.08	**31.1**	0.65	–	–	10.0	Y
c11	24–27 Nov	43.0	1.17	**29.6**	0.61	34.0	0.61	13.3	Y
c12	28–2 Nov	45.7	1.31	**25.0**	0.50	31.8	0.55	20.7	Y
c13	3–7 Dec	**39.7**	1.02	40.0	0.93	–	–	−0.3	N
c14	8–11 Dec	45.0	1.27	**37.2**	0.83	–	–	7.8	Y
c15	12–15 Dec	48.2	1.46	**40.0**	0.93	45.8	1.00	8.3	Y
c16	16–19 Dec	50.0	1.57	**45.8**	1.19	–	–	4.2	Y
c17	20–23 Dec	47.9	1.44	51.6	1.50	**27.2**	0.49	20.8	Y
c18	24–28 Dec	39.6	1.02	**33.6**	0.72	36.8	0.68	6.0	Y
c19	29–1 Jan	52.1	1.71	**29.7**	0.61	32.5	0.57	21.0	Y
c20	2–6 Jan	54.6	1.90	**24.5**	0.49	–	–	30.1	Y
c21	7–10 Jan	48.4	1.47	**13.8**	0.31	19.4	0.33	34.6	Y
c22	11–14 Jan	47.2	1.40	**35.0**	0.76	41.2	0.82	12.2	Y
*unspecked hardyhead*									
c1	10–13 Oct	–	–	–	–	–	–	–	–
c2	14–17 Oct	–	–	–	–	–	–	–	–
c3	18–22 Oct	–	–	–	–	–	–	–	–
c4	23–27 Oct	–	–	–	–	–	–	–	–
c5	28–2 Oct	–	–	–	–	–	–	-	–
c6	3–7 Nov	–	–	–	–	–	–	–	–
c7	8–11 Nov	–	–	–	–	–	–	–	–
c8	12–15 Nov	20.3	0.48	–	–	**17.5**	0.31	2.8	N
c9	16–19 Nov	23.6	0.55	69.9	0.50	**22.2**	0.39	1.4	N
c10	20–23 Nov	**24.4**	0.58	65.5	0.47	–	–	−41.1	N
c11	24–27 Nov	**31.0**	0.78	92.9	0.68	35.8	0.72	–4.8	N
c12	28–2 Nov	36.4	1.00	–	–	**34.6**	0.68	1.7	N
c13	3–7 Dec	26.7	0.64	–	–	–	–	–	?
c14	8–11 Dec	25.3	0.60	–	–	–	–	–	?
c15	12–15 Dec	**16.0**	0.39	48.0	0.37	17.4	0.31	–1.4	N
c16	16–19 Dec	**19.0**	0.45	65.5	0.47	–	–	–46.5	N
c17	20–23 Dec	**25.3**	0.60	–	–	27.2	0.49	−1.9	N
c18	24–28 Dec	**31.2**	0.78	–	–	34.8	0.69	–3.6	N
c19	29–1 Jan	**36.8**	1.00	108.7	0.83	39.9	0.87	−3.1	N
c20	2–6 Jan	**32.7**	0.84	112.8	0.88	–	–	−80.1	N
c21	7–10 Jan	**34.4**	0.91	–	–	42.9	1.00	−8.5	N
c22	11–14 Jan	**40.3**	1.19	135.8	1.19	–	–	−95.5	N

Bold = model of best fit. CP  =  critical period, where Y = yes, N = no,? =  could not be determined.

### Age-specific mortality rates

**Figure 5:**
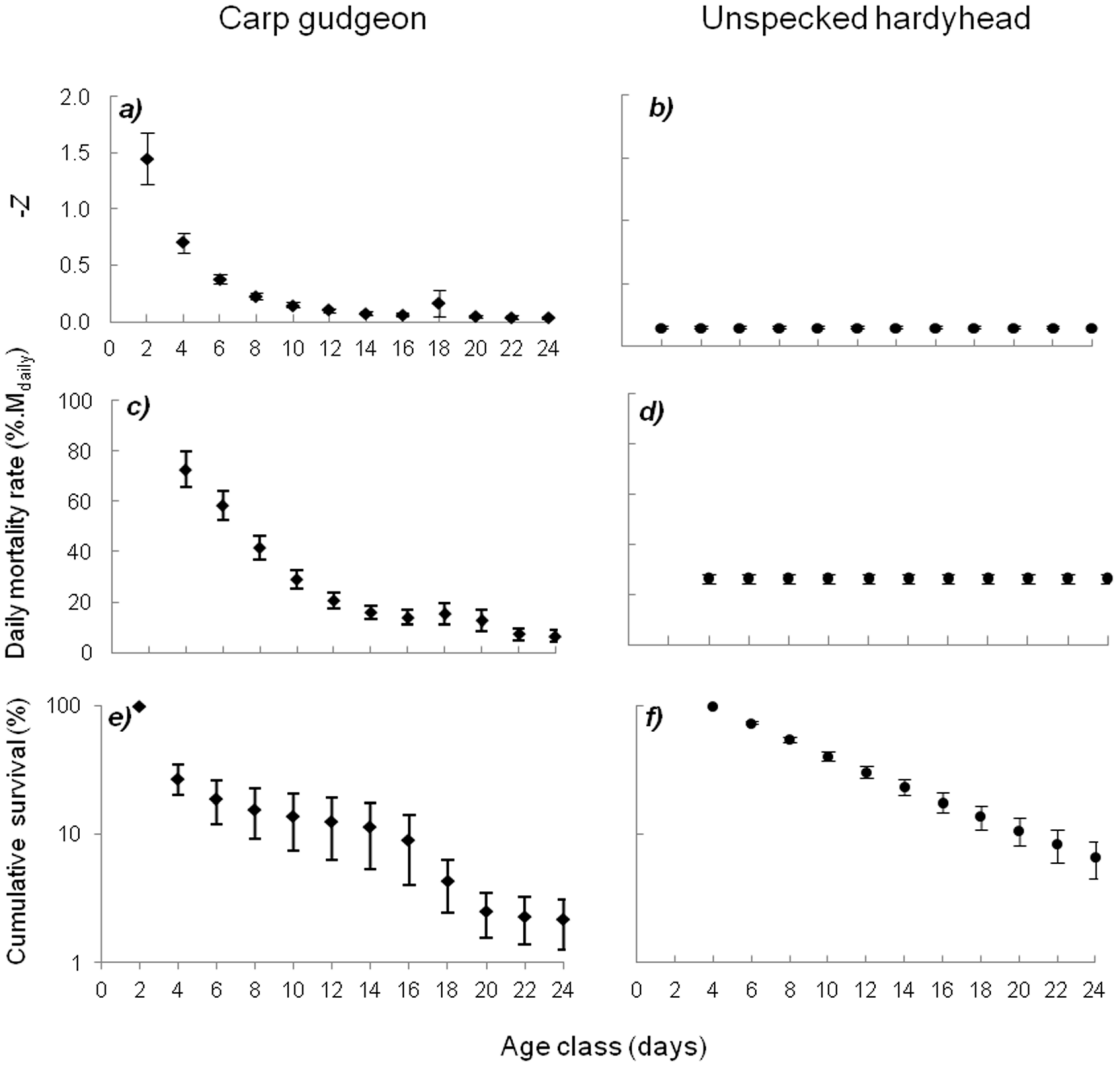
Mean (±SE) cohort; instantaneous mortality rates (-*Z*), daily mortality rates (M_daily_) and cumulative survival (%) of carp gudgeon and unspecked hardyhead larvae. Age class (days) represent 2 day groupings of larvae (e.g. 2 = 1–2 day old larvae, 4 = 3–4 day old larvae etc).

Mean cohort age-specific mortality rates for all cohorts analysed for carp gudgeon and unspecked hardyhead revealed contrasting patterns between the two species (Figure 5). Mean carp gudgeon instantaneous mortality rates (-*Z*) peaked at 1.4 (max  =  3.8) for 1–2 day-old larvae, at which time they declined exponentially with age, to 0.03 at 23–24 days old (min <0.01) (Figure 5*a*). Mean -*Z* for unspecked hardyhead cohorts was constant across all ages, with a mean of 0.15 (max = 0.20, Figure 5*b*). Mean -*Z* was lower in unspecked hardyhead than carp gudgeon early in development, but beyond 9-10 days, hardyhead mortality rates exceeded those of carp gudgeon until metamorphosis. Variation around mean -*Z* across the cohorts was also more pronounced in carp gudgeon than in unspecked hardyhead.

These patterns were also reflected in daily mortality rates, with the highest daily mortality rates experienced by carp gudgeon at 3–4 (73 %loss.d^−1^), 5–6 (58 %loss.d^−1^) and 7–8 (42 %loss.d^−1^) days (Figure 5*c*). By 21–22 days old, daily mortality rates were less than 10 %loss.d^−1^. Hardyhead daily mortality rates were 26.4 %loss.d^−1^ regardless of age or developmental stage. (Figure 5*d*). Mean cumulative mortality for carp gudgeon for the entire larval phase was 97.8 ± 0.9 % (Figure 5*e*) and for unspecked hardyhead larvae from 3–4 until 24 days old was 93.3 ± 2.2 % (Figure 5*f*). Based on estimated numbers of 1–2 day old larvae, overall mortality for unspecked hardyhead for the entire larval period was estimated to be 94.3%.

## Discussion

Our results show clear evidence of a critical period – accelerated age-specific mortality rates at one particular time of larval development - for one species of small, protracted-spawning riverine fish. Twenty-one of 22 larval cohorts of carp gudgeon showed that non-linear mortality curves best fit changes in abundance with age. Mortality rates were greatest for larvae up to 6 days old, which coincided with the timing of first exogenous feeding. In contrast, there was no evidence to support a critical period in unspecked hardyhead for any of the cohorts followed.

The critical period hypothesis, and its purported link to the switch from endogenous to exogenous feeding, is a central tenet of recruitment ecology of fish. Yet despite this, few previous studies have sampled the early life stages of fish at appropriate temporal and spatial scales to test this hypothesis effectively [6], [12]. Those studies that have followed individual cohorts through the majority of their larval phase have found evidence of critical periods in some species, such as American shad, *Alosa sapidissima* [38], freshwater dace, *Leuciscus leuciscus* [39], but not in freshwater drum, *Aplodinotus gruniens* [40], pike, *Esox lucius* [41], white crappie, *Pomoxis annularis*, or black crappie, *Pomoxis nigromaculatus* [42]. A lack of a suitable, standardised methodology and analytical approach for testing this hypothesis makes comparisons across species difficult. In the present study, the pronounced inflexion of the survivorship curves of carp gudgeon closely conforms to a non-linear model, so can be described unequivocally as having a critical period, whilst the survivorship curves of unspecked hardyhead showed no evidence.

Differences in early life history traits may explain the presence or absence of critical periods in carp gudgeon and unspecked hardyhead. Here, we hypothesise that two traits i) the presence/absence of overlap between endogenous feeding; and ii) development state and size at hatch may play a role in the species-specific patterns observed.

For fish to begin feeding exogenously, they must learn how to detect, strike and digest their food [43], [44]. Prey capture success has been shown to improve significantly within days of the switch from endogenous to exogenous feeding for a variety of species [45], [46]. It follows that higher mortality rates are expected at the onset of this learning phase [47], [48] and this appeared to be the case for carp gudgeon in the present study. There have been few studies which have investigated the development of carp gudgeon or unspecked hardyhead around the time of transition from endogenous to exogenous feeding [26], [29]. For carp gudgeon, however, previous observations made on spawning events in aquaria, and gut contents in wild fish, suggest that yolksac reserves are exhausted prior to the commencement of feeding exogenously [25]. In the present study, carp gudgeon generally retained their yolksac for the first 1–2 days post hatch. With the exception of those sampled in October, by the third day, the majority of individuals had exhausted their yolksac, but remained as protolarvae until 6–8 days of age. By contrast, the onset of exogenous feeding occurs prior to the exhaustion of yolksac reserves in unspecked hardyhead. In this species, a transitional overlap between endogenous and exogenous feeding appears to last for 3–5 days, before larvae begin feeding solely on external food sources [29]. Thus, because their early survival is less dependent on the success of early, inefficient attempts at prey capture, hardyhead larvae may be more likely to experience lower mortality rates than carp gudgeon under the same environmental conditions. The observed difference in the mortality rates around the time of exogenous feeding supports this prediction.

Differences in developmental state at hatch may also contribute to the higher mortality rates of carp gudgeon than unspecked hardyhead. At hatch, carp gudgeon larvae are small (1.5 – 2.1 mm) and poorly developed. Larvae are transparent, their eyes unpigmented, jaws undeveloped and incapable of feeding, and their pectoral fins have not yet formed [49]. Unspecked hardyhead larvae, on the other hand, are much larger at hatch (approximately 5 mm), they are well developed, their eyes are pigmented, and the jaws are ossified and fully functioning [29], making them more likely to be successful at capturing and ingesting prey at an earlier age. As a result, unspecked hardyhead may be less vulnerable to starvation during the time of first feeding than carp gudgeon.

The effect of size of individuals on mortality rates has also been well documented for a variety of organisms, including fish [14], birds [50], mammals [51], and invertebrates [52]. For fish, larger conspecifics have generally been shown to have lower energy demands per unit mass [53], and have proportionally greater energy stores [54]. Swimming ability, which for larval fish is advantageous for predator avoidance [55] and prey capture success [56] also increases with body size. Previous studies investigating the role of size at hatch on early larval dynamics have also shown that fish that are larger at hatch take longer to absorb their yolksac, are able to commence exogenous feeding earlier, and take longer to reach irreversible starvation than do those species that are smaller at hatch [57]. Furthermore, Fuiman and Werner (2002) [58] suggest that species that produce small eggs do not have the advantage of large, well-developed larvae at hatching. Instead, such species improve their chances of having offspring that survive through a reproductive age by producing more eggs. It is therefore axiomatic that species such as carp gudgeon, that produce large numbers of comparatively small eggs and are poorly developed at hatch, would be expected to experience greater mortality over the first few days post-hatch than species like unspecked hardyhead, which are less fecund but produce larger eggs.

The contrasting mortality patterns of the larval phases of the two species studied, highlight the potential role that early life history traits play, not just as drivers of total [23], but in shaping the pattern of, mortality experienced during the larval phase. We hypothesise that species which produce relatively large numbers of small eggs and are poorly developed at hatch, are likely to experience greater mortality over the first few days post-hatch than species which are relatively less fecund but produce larger eggs. While our study here reports only on two species, further research looking at a wider range of species along a gradient of life history traits is warranted.

## Supporting Information

Appendix S1 **Validation of daily increment formation in otoliths for carp gudgeon and unspecked hardyhead larvae and juveniles.**
(DOC)

Appendix S2 **Monthly age-length relationships for carp gudgeon and unspecked hardyhead during their larval and early juvenile phases using von Bertalanffy, Gompertz and linear growth models.**
(DOC)

Appendix S3 **Model parameters for individual cohort and population based carp gudgeon and unspecked hardyhead survival curves.**
(DOC)

Appendix S4 **Size selectivity of modified-quatrefoil light traps.**
(DOC)
