# Supplementary material for: Contrasting Patterns of Larval Mortality in Two Sympatric Riverine Fish Species: A Test of the Critical Period Hypothesis
Source: PLoS One. 2014 Oct 9;9(10):e109317. doi: 10.1371/journal.pone.0109317 (PMC4192135; doi:10.1371/journal.pone.0109317)
Supplement: Appendix S1 — Validation of daily increment formation in otoliths for carp gudgeon and unspecked hardyhead larvae and juveniles. (DOC) [file pone.0109317.s001.doc]

Appendix S1: Validation of daily increment formation in otoliths for carp gudgeon (*Hypseleotris* spp.) and unspecked hardyhead (*Craterocephalus stercusmasacarum fulvus*) larvae and juveniles.

*Methods*

Larvae were collected from the Lindsay River on the night of 17 January 2009. Eight light traps were randomly deployed along the littoral zone of a 600 m of river and left for approximately 2 h (22:00-00:00 h). Light traps were retrieved carefully by placing them in a water filled container prior to being pulled out of the river, making sure that larvae were not damaged during the handling process. Larvae were immediately transported in aerated plastic tanks containing filtered (53 µm) river water to the laboratory, where carp gudgeon and unspecked hardyhead larvae and juveniles were live picked using a dissecting microscope and plastic pipette. Individuals were randomly assigned to one of two Alizarin Red® baths: a short term exposure bath and a long term exposure bath. The short term exposure bath had a buffered concentration of 1 g.L^-1^ Alizarin Red and 5 g.L^-1^ Sodium Chloride (pH 7.5), in which larvae were exposed for 3-5 minutes under complete darkness. The long term exposure bath had a buffered concentration of 300 mg.L^-1^ Alizarin Red and 1 g.L^-1^ Sodium Chloride (pH 7.5). In this treatment, larvae were left in the baths for 12 h under complete darkness. Once larvae had been exposed to either treatment, they were transferred into one of two aerated 20L glass aquaria were they remained outside for a further 8 days under natural light conditions (14 h light: 10 h dark photoperiod). Larvae were fed daily with rotifers and zooplankton which had been collected from a nearby wetland using a 5 m hand trawl net (53*µ*m mesh). Larvae were retrieved from the tanks at the end of the experiment and immediately preserved in 90% ethanol.

Individual larvae were placed directly onto glass slides. Using a dissecting microscope and pins, the left sagittal otolith of each larvae was removed and mounted onto the slide with epoxy resin. Otoliths were polished and read using a fluorescent microscope. Otoliths were checked for fluorescing rings, and the number of rings deposited between this point check and the outer most ring were counted. If the number of rings matched the number of days lapsed during the field experiment (± 1day), the daily deposition of otolith rings could be confirmed. All otoliths were read twice. If there was discrepancy within the two readings by more than ± 2 days, the otolith was discarded. If there was a discrepancy within the two readings by ±1 day, a third count was made by an independent reader. Readings made under 40x – 100x magnifications, using immersion oil.

*Results*

A total of 258 carp gudgeon and 82 unspecked hardyhead larvae and juveniles were collected and exposed to the two Alizarin baths. High rates of mortality occurred in the both the short and long term exposure treatments to Alizarin Red. Twelve hours post emersion, only 74 carp gudgeon and 39 unspecked hardyhead larvae/juveniles were alive. Of these, the otoliths of 12 carp gudgeon and eight unspecked hardyheads exhibited fluorescing rings. All of the otoliths that were stained successfully exhibited eight countable increments between the outermost ring and the fluorescing ring (Figure S1). The biggest carp gudgeon larvae/juvenile successfully stained was 83 mm (TL, juvenile stage). The biggest unspecked hardyhead larvae/juvenile successfully stained was 101 mm (TL, juvenile stage). The time between marking and the experiment terminating was 8 days, indicating that increment formation was a daily event for carp gudgeon and unspecked hardyhead larvae and early juveniles.

**Alizarin stain under UV light**

**Daily increment formation**

*a)* carp gudgeon

*b)* Unspecked hardyhead

**Figure S1:** Photographs of increment deposition on the sagittal otoliths of *a)* carp gudgeon and *b)* unspecked hardyhead larvae as viewed under bright field, and their associated Alizarin marks as viewed under UV light.
