# Supplementary material for: Contrasting Patterns of Larval Mortality in Two Sympatric Riverine Fish Species: A Test of the Critical Period Hypothesis
Source: PLoS One. 2014 Oct 9;9(10):e109317. doi: 10.1371/journal.pone.0109317 (PMC4192135; doi:10.1371/journal.pone.0109317)
Supplement: Appendix S2 — Monthly age-length relationships for carp gudgeon and unspecked hardyhead during their larval and early juvenile phases using von Bertalanffy, Gompertz and linear growth models. (DOC) [file pone.0109317.s002.doc]

Appendix S2: Monthly age-length relationships for carp gudgeon and unspecked hardyhead during their larval and early juvenile phases using von Bertalanffy, Gopertz and linear growth models.

**Figure S2:** Monthly age-length relationships for carp gudgeon and unspecked hardyhead during their larval and early juvenile phases for *a)* October, *b)* November, *c)* December and *d)* January. Growth models fitted to the age-length data are von Bertalanffy (dotted line), Gompertz (dashed line), and linear (solid line). Parameters for gompertz model^1^ included.

^1^The Gompertz growth function was described by the equation:

$$L_{i}=L_{\infty}e^{-e[-k(t_{i}-t_{0})]}$$

where L*_i_* is the expected length at age for the *i*th individual, L_∞_ is the theoretical maximum length, *k* is the Brody growth co-efficient, *t*_0_ is the theoretical age at length 0, and *t_i_* is the true age of the *i*th individual.

**Table S2:** Adjusted R^2^ and AIC results of fitted monthly growth equations for carp gudgeon and unspecked hardyhead.

|  |  | | Carp gudgeon | | | | | |  | | Unspecked hardyhead | | | | | |  |
| --- | --- | --- | --- | --- | --- | --- | --- | --- | --- | --- | --- | --- | --- | --- | --- | --- | --- |
|  | No. of model parameters | | SE | | Adj. R^2^ | | AIC | |  | | SE | | Adj. R^2^ | | AIC | |  |
|  | |  | |  | |  | |  | |  | |  | |  | |  | |
| *October* | |  | | *(n=24)* | |  | |  | |  | | *(n=27)* | |  | |  | |
| Linear | | 1 | | 0.498 | | 0.961 | | 38.55 | |  | | 0.912 | | 0.683 | | **75.58** | |
| Gompertz | | 3 | | 0.412 | | 0.970 | | **30.37** | |  | | 0.931 | | 0.983 | | 77.59 | |
| von Bertalanffy | | 3 | | 0.415 | | 0.974 | | 30.72 | |  | | - | | - | | - | |
|  | |  | |  | |  | |  | |  | |  | |  | |  | |
| *November* | |  | | *(n=26)* | |  | |  | |  | | *(n=29)* | |  | |  | |
| Linear | | 1 | | 0.347 | | 0.979 | | 22.60 | |  | | 0.458 | | 0.969 | | 40.95 | |
| Gompertz | | 3 | | 0.287 | | 0.997 | | 13.70 | |  | | 0.419 | | 0.993 | | **36.70** | |
| von Bertalanffy | | 3 | | 0.284 | | 0.986 | | **13.10** | |  | | - | | - | | - | |
|  | |  | |  | |  | |  | |  | |  | |  | |  | |
| *December* | |  | | *(n=26)* | |  | |  | |  | | *(n=31)* | |  | |  | |
| Linear | | 1 | | 0.594 | | 0.965 | | **50.63** | |  | | 0.449 | | 0.959 | | **42.23** | |
| Gompertz | | 3 | | 0.625 | | 0.958 | | 54.17 | |  | | 0.453 | | 0.997 | | 43.71 | |
| von Bertalanffy | | 3 | | 0.604 | | 0.966 | | 52.39 | |  | | 0.455 | | - | | 44.01 | |
|  | |  | |  | |  | |  | |  | |  | |  | |  | |
| *January* | |  | | *(n=29)* | |  | |  | |  | | *(n=34)* | |  | |  | |
| Linear | | 1 | | 0.677 | | 0.967 | | 63.63 | |  | | 0.688 | | 0.878 | | 74.96 | |
| Gompertz | | 3 | | 0.686 | | 0.963 | | 65.26 | |  | | 0.453 | | 0.997 | | **43.71** | |
| von Bertalanffy | | 3 | | 0.656 | | 0.970 | | **62.72** | |  | | - | | - | | - | |
|  | |  | |  | |  | |  | |  | |  | |  | |  | |
