# Supplementary material for: Contrasting Patterns of Larval Mortality in Two Sympatric Riverine Fish Species: A Test of the Critical Period Hypothesis
Source: PLoS One. 2014 Oct 9;9(10):e109317. doi: 10.1371/journal.pone.0109317 (PMC4192135; doi:10.1371/journal.pone.0109317)
Supplement: Appendix S3 — Model parameters for individual cohort and population based carp gudgeon and unspecked hardyhead survival curves. (DOC) [file pone.0109317.s003.doc]

**Appendix S3: Model parameters for four-day cohort and population based carp gudgeon and unspecked hardyhead survival curves**

Table S3: Model parameters for the best survival curve for each 4 day carp gudgeon and unspecked hardyhead cohort. Models constructed with log_e_(x+1) transformed data. Model = model of best fit as determined by AICc scores, where L=linear^1^, A=asymptotic^2^ and W=Weibull^3^  functions. See footnotes for model equations and parameter definitions.

| Cohort no. | Hatch date | | Model | | *Z* | | *a* | | | *R_o_* | | | *lrc* | | | | | *d* | *pwr* | | |
| --- | --- | --- | --- | --- | --- | --- | --- | --- | --- | --- | --- | --- | --- | --- | --- | --- | --- | --- | --- | --- | --- |
| *Carp gudgeon* | | | | | |  | |  | | |  | | |  | |  | | | |  | |
| c1 | 10-13 Oct | | A | | - | | 2.12 | | | 20.30 | | | -0.13 | | | | | - | - | | |
| c2 | 14-17 Oct | | - | | - | | - | | | - | | | - | | | | | - | - | | |
| c3 | 18-22 Oct | | L | | -0.13 | | - | | | 5.46 | | |  | | | | | - | - | | |
| c4 | 23-27 Oct | | L | | -0.18 | | - | | | 6.39 | | | - | | | | | - | - | | |
| c5 | 28-2 Nov | | A | | - | | 3.91 | | | 10.81 | | | -1.48 | | | | | - | - | | |
| c6 | 3-7 Nov | | W | | - | | 8.33 | | | - | | | 7.25 | | | | | 4.39 | -5.45 | | |
| c7 | 8-11 Nov | | A | | - | | 3.65 | | | 12.26 | | | -0.73 | | | | | - | - | | |
| c8 | 12-15 Nov | | W | | - | | 1.98 | | | - | | | -37.36 | | | | | -1.67 | 13.25 | | |
| c9 | 16-19 Nov | | A | | - | | 1.15 | | | 9.22 | | | -2.05 | | | | | - | - | | |
| c10 | 20-23 Nov | | A | | - | | 0.86 | | | 10.82 | | | -1.77 | | | | | - | - | | |
| c11 | 24-27 Nov | | A | | - | | 1.76 | | | 10.97 | | | -1.42 | | | | | - | - | | |
| c12 | 28-2 Dec | | A | | - | | 2.03 | | | 12.76 | | | -1.29 | | | | | - | - | | |
| c13 | 3-7 Dec | | L | | -0.23 | | - | | | 6.21 | | | - | | | | | - | - | | |
| c14 | 8-11 Dec | | A | | - | | 1.49 | | | 10.75 | | | -1.46 | | | | | - | - | | |
| c15 | 12-15 Dec | | A | | - | | 1.51 | | | 12.20 | | | -1.22 | | | | | - | - | | |
| c16 | 16-19 Dec | | A | | - | | 1.71 | | | 17.62 | | | -0.82 | | | | | - | - | | |
| c17 | 20-23 Dec | | W | | - | | 3.97 | | | - | | | 118.99 | | | | | 2.92 | -41.15 | | |
| c18 | 24-28 Dec | | A | | - | | -0.75 | | | 9.34 | | | -2.31 | | | | | - | - | | |
| c19 | 29-1 Jan | | A | | - | | 0.06 | | | 13.53 | | | -1.34 | | | | | - | - | | |
| c20 | 2-6 Jan | | A | | - | | 0.27 | | | 21.06 | | | -0.67 | | | | | - | - | | |
| c21 | 7-10 Jan | | A | | - | | 0.22 | | | 15.71 | | | -0.85 | | | | | - | - | | |
| c22 | 11-14 Jan | | A | | - | | 0.85 | | | 11.62 | | | -0.86 | | | | | - | - | | |
|  | |  | |  | |  | |  | | |  | | |  | |  | | | |  | |
| *Unspecked hardyhead* | | | | | | | | |  | | |  | | |  | |  | | | |  |
| c8 | 12-15 Nov | | L | | -0.10 | | - | | | 4.44 | | | - | | | | | - | - | | |
| c9 | 16-19 Nov | | L | | -0.18 | | - | | | 5.40 | | | - | | | | | - | - | | |
| c10 | 20-23 Nov | | L | | -0.19 | | - | | | 5.18 | | | - | | | | | - | - | | |
| c11 | 24-27 Nov | | L | | -0.13 | | - | | | 4.13 | | | - | | | | | - | - | | |
| c12 | 28-2 Dec | | L | | -0.06 | | - | | | 3.61 | | | - | | | | | - | - | | |
| c13 | 3-7 Dec | | - | | - | | - | | | - | | | - | | | | | - | - | | |
| c14 | 8-11 Dec | | - | | - | | - | | | - | | | - | | | | | - | - | | |
| c15 | 12-15 Dec | | L | | -0.12 | | - | | | 5.26 | | | - | | | | | - | - | | |
| c16 | 16-19 Dec | | L | | -0.18 | | - | | | 5.81 | | | - | | | | | - | - | | |
| c17 | 20-23 Dec | | L | | -0.19 | | - | | | 5.77 | | | - | | | | | - | - | | |
| c18 | 24-28 Dec | | L | | -0.19 | | - | | | 5.82 | | | - | | | | | - | - | | |
| c19 | 29-1 Jan | | L | | -0.20 | | - | | | 4.96 | | | - | | | | | - | - | | |
| c20 | 2-6 Jan | | L | | -0.13 | | - | | | 3.92 | | | - | | | | | - | - | | |
| c21 | 7-10 Jan | | L | | -0.15 | | - | | | 4.00 | | | - | | | | | - | - | | |
| c22 | 11-14 Jan | | L | | -0.17 | | - | | | 4.94 | | | - | | | | | - | - | | |

*Footnotes:*

^1^The linear regression model was described as:

where *Z*=rate of mortality, R_0_=intercept

^2^The asymptotic regression model was described as:

where a=asymptote, R_0_= when x=0, lrc=log(rate constant).

^3^The Weibull regression model was described as:

where a=asymptote, d=drop, lrc=log(rate constant), and pwr=numeric constant.
