# Supplementary material for: Contrasting Patterns of Larval Mortality in Two Sympatric Riverine Fish Species: A Test of the Critical Period Hypothesis
Source: PLoS One. 2014 Oct 9;9(10):e109317. doi: 10.1371/journal.pone.0109317 (PMC4192135; doi:10.1371/journal.pone.0109317)
Supplement: Appendix S4 — Size selectivity of modified-quatrefoil light traps. (DOC) [file pone.0109317.s004.doc]

**Appendix S4: Size selectivity of modified-quatrefoil light traps**

Introduction

When investigating population dynamics, it is important to determine whether the sampling method influences the size of the fish captured. For example, drift nets, tow nets and bongo nets have found to be size-selective methods: early stage larvae tend to be under-represented due to their small size, whilst many juvenile fish are also under-represented due to their ability to actively avoid nets (Kelso and Rutherford 1996). The aim of this experiment was to investigate size selectivity of modified quatrefoil light traps (encased in 3 mm mesh) in collecting the larvae of carp gudgeon *Hypseleotris* sp. and unspecked hardyhead *Craterocephalus stercusmusarum fulvus*.

Methods

A mesocosm experiment was conducted in the Lindsay River from 14 – 15 January 2009. Six 1.5 x 1.5 x 0.4 m inflatable, pre-conditioned, plastic wading pools were set up in the Lindsay River, Victoria, Australia (517178E, 6219649N). Mesocosms where filled with filtered (<53 *µ*m) river water to a depth 0.3 m. Larval and juvenile carp gudgeon and unspecked hardyhead larvae were collected from the Lindsay River on 14 January 2009 using hand trawls, light traps without mesh, and larval beach seines. It was considered important to use a combination of sampling methods to collect a wide size range of larvae and juvenile fish. Hand trawls involved throwing a 30 cm diameter, 250 *µ*m conical net 10 m into the pelagic zone of the river, and pulling it back through the water column. Additionally, multiple 250 mm x 4 m long x 1 m deep beach seines were pulled through the littoral area of the river. Twelve quatrefoil light traps set with yellow 12 h Cyalume® light sticks were deployed along the littoral edge of the Lindsay River for 2 h at night. All individuals collected were immediately cleared from the gear and placed in a 20 L aerated holding aquaria.

After the sampling collection had finished, larvae in the holding aquaria were randomly and evenly distributed amongst the 6 mesocosms. That evening, after dusk, a quatrefoil light trap wrapped in 3 mm mesh and containing a yellow Cyalume® light stick was placed in the middle of each mesocosm and left for 1.5 h. After the 1.5 h period, larvae collected in the light traps were euthanased and immediately preserved in 70% ethanol. Any larvae which had not been collected by the light traps and which remained in the mesocosm were collected by draining the water with a bilge pump through a 50 *µ*m mesh net that had a reducing jar attached. The net was rinsed, and the entire sample preserved immediately in 70% ethanol. There was no mortality caused by handling.

Data analysis

All larvae from the mesocosms were identified, staged and measured (TL mm) to 0.1 mm resolution using a dissecting microscope. To estimate the ability of individuals to pass through the mesh of the mesocosms, maximum heights (mm) of all fish in the experiment were measured. Height measurements were taken as the diameter of the individual from the widest point, which most commonly occurred at the pectoral fins. The sizes of the larval and juvenile carp gudgeon and hardyhead collected from the 6 light traps was compared to the initial larval population contained in the mesocosms at the commencement of the study.

Results & Discussion

A total of 233 carp gudgeon and 102 hardyhead larvae and juveniles were collected from the Lindsay River and used in the study. Light traps successfully caught 100% of all carp gudgeon larvae (n=119) and unspecked hardyhead larvae (n=12) from the 6 mesocosms (Figure S4), indicating there was no size-selectivity occurring for either species during their larval phase. Evidence of size selective behaviour commenced well into the juvenile phase of carp gudgeon; the maximum length of a carp gudgeon larvae recorded was 12.1 TL mm, whilst the smallest fish not collected by the light traps was 15.0 mm TL (Figure S4*a*). Only a very small number of hardyhead larvae could be found in the Lindsay River during the sampling period, and with very few late- staged larvae collected. However, results of the mesocosm study showed that hardyheads had all reached juvenile stage by 10.6 mm TL, and that light traps only became less effective in trapping individuals once they had reached > 12 mm TL (Figure S4*b*).

Max. height (mm)

*a)* Carp gudgeon (*n*=233)

*b)* Unspecked hardyhead (*n*=102)

Length (TL mm)

**Figure S4:** Size selectivity of modified quatrefoil light-traps wrapped with 3 mm mesh; *a)* regression of carp gudgeon length (TL) vs. maximum height, *b)* regression of unspecked hardyhead length (TL) vs. maximum height. Grey dashed ellipses encompass larval phase of fish. (o) = individuals collected in light traps, (+) = individuals not captured by light traps.
